# Rab5ab-Mediated Yolk Cell Membrane Endocytosis Is Essential for Zebrafish Epiboly and Mechanical Equilibrium During Gastrulation

**DOI:** 10.3389/fcell.2021.697097

**Published:** 2021-10-29

**Authors:** Maria Marsal, Amayra Hernández-Vega, Philippe-Alexandre Pouille, Enrique Martin-Blanco

**Affiliations:** Instituto de Biología Molecular de Barcelona, Consejo Superior de Investigaciones Científicas, Parc Cientific de Barcelona, Barcelona, Spain

**Keywords:** endocytosis, zebrafish, Rab5, epiboly, mechanics

## Abstract

Morphogenesis in early embryos demands the coordinated distribution of cells and tissues to their final destination in a spatio-temporal controlled way. Spatial and scalar differences in adhesion and contractility are essential for these morphogenetic movements, while the role that membrane remodeling may play remains less clear. To evaluate how membrane turnover modulates tissue arrangements we studied the role of endocytosis in zebrafish epiboly. Experimental analyses and modeling have shown that the expansion of the blastoderm relies on an asymmetry of mechanical tension in the yolk cell generated as a result of actomyosin-dependent contraction and membrane removal. Here we show that the GTPase Rab5ab is essential for the endocytosis and the removal of the external yolk cell syncytial layer (E-YSL) membrane. Interfering in its expression exclusively in the yolk resulted in the reduction of yolk cell actomyosin contractility, the disruption of cortical and internal flows, a disequilibrium in force balance and epiboly impairment. We conclude that regulated membrane remodeling is crucial for directing cell and tissue mechanics, preserving embryo geometry and coordinating morphogenetic movements during epiboly.

## Introduction

Cell rearrangements preserve the cohesion of tissues and the integrity of the embryo in multiple morphogenetic processes. These rearrangements associate to cells shape changes and cell-cell contacts that result from the redistribution of apical, basal and lateral surfaces without volume changes (Lee and Harland, 2010; Mateus et al., 2011; Fabrowski et al., 2013). Despite the relevance of surface area redistribution for morphogenesis we still know little on how it is regulated. Expansion and reduction of a given plasma membrane surface area are regulated by exocytosis and endocytosis (Lecuit and Pilot, 2003; Lee and Harland, 2010). Endocytosis, in particular, has been shown to be important for cells apical constriction [reviewed in Doherty and McMahon (2009), affecting tension and contractility (Betchaku and Trinkaus, 1986; Gauthier et al., 2012). It affects distinct events, ranging from cell specific, (Satoh et al., 2008)] to large-scale morphogenetic processes [reviewed in Doherty and McMahon (2009)]. Cellularization and dorsal closure in *Drosophila* and neurulation and apical constriction of bottle cells during gastrulation in *Xenopus laevis* are just some examples (Lee and Harland, 2010; Mateus et al., 2011; Fabrowski et al., 2013). In the zebrafish, endocytosis affects Silberblick (slb)/Wnt11 activity and E-cadherin trafficking, necessary for epiboly (Song et al., 2013) and the coordinated movement of the prechordal plate (Ulrich et al., 2005).

Endocytosis can be clathrin-dependent, caveolae-mediated or via macropinocytosis. Clathrin-mediated endocytosis (CME), a mechanism for controlled cargo uptake, is the most general membrane internalization route. It is characterized by the formation of clathrin-coated vesicles (CCV) and the selective internalization of cell-surface components and extracellular macromolecules [for review see McMahon and Boucrot (2011)]. CME depends on Dynamin GTPase activity for vesicle budding and scission, which leads to trafficking through the endocytic pathway upon clathrin coat disassembling. Caveolae (which incorporate caveolin) are also widely employed by cells for membrane removal. They associate to cholesterol pools and are involved in the internalization of specific markers and perhaps in transcytosis. Last, internalization can also take place by macropinocytosis, which usually occurs within highly ruffled regions of the plasma membrane and is involved in large-scale membrane internalization (Swanson and Watts, 1995; Cao et al., 2007; Lim and Gleeson, 2011).

**FIGURE 1:**
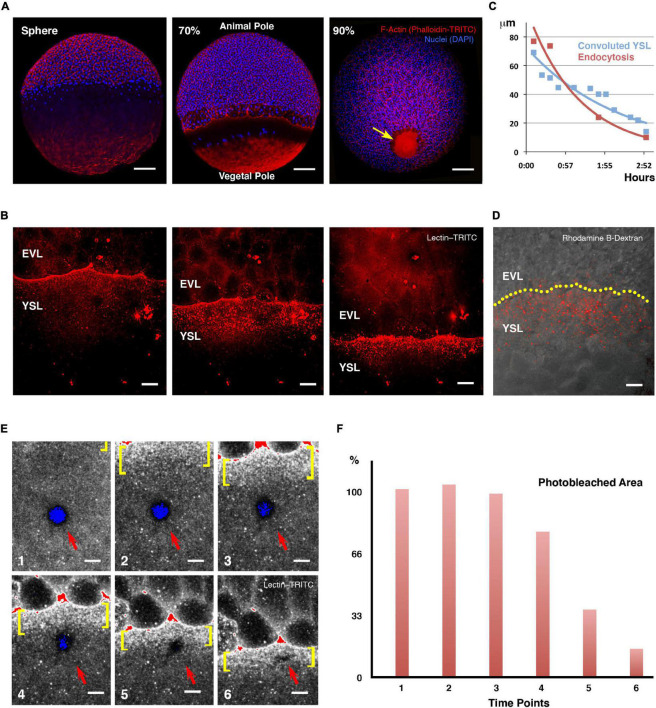
Yolk cell membrane endocytosis at the E-YSL. **(A)** Blastoderm expansion during epiboly. At sphere stage epiboly has not yet begun (left). F-actin accumulates at the periphery of all cells as well as in the yolk cell, mainly at the vegetal cap. At 70% epiboly (middle), the blastoderm has crossed the equator and will decrease its margin until closure. A belt of actin develops at the E-YSL ahead of the EVL and an actin-free zone separates this belt from a vegetal actin-rich patch. At 90% epiboly (right), the E-YSL and the vegetal actin-rich patch merge at the vegetal pole (arrow). Embryos were stained with phalloidin-TRITC (red) and DAPI (blue). Scale bars 100 μm. **(B)** Sequential images of a confocal time-lapse video of a wild type embryo soaked in lectin–TRITC for 5 min at sphere stage. The lectin binds to the membrane of both the yolk cell and the EVL cells and gets internalized accumulating in vesicles in the E-YSL just ahead of the EVL margin (from Supplementary Movie 1). EVL and YSL are indicated. Scale bar 25 μm. All confocal images are maximum projections. **(C)** Parallel reduction of the width of the convoluted E-YSL domain (blue) and the area undergoing membrane removal (red) during epiboly progression. X and Y axes represent hours after 50% epiboly and width in μm, respectively. **(D)** Uptake of fluorescent dextran (red) at the E-YSL just ahead of the EVL margin (yellow dots) at 65% epiboly. Scale bar 25 μm. **(E)** Snapshots of time-lapse images (from Supplementary Movie 2) of a lectin-TRITC soaked embryo (levels color coded as a range indicator) showing a circular photobleached area (red arrow) in the yolk cell away from the EVL leading edge. The photobleached membrane is removed and endocytosed only upon its enclosure within the advancing E-YSL (yellow brackets). All confocal images are maximum projections. Scale bar 25 μm. **(F)** Membrane internalization dynamics. Histograms depicting the percentage of the photobleached area reduction (red) at each regular sequential time points in **(E)**. The removal of the photobleached membrane initiates once becomes a part of the convoluted area ahead of the leading front (from time 4 onward).

We aimed to understand the role that membrane removal plays during the conserved early morphogenetic movements leading to epiboly in the zebrafish. At the onset of zebrafish epiboly (sphere stage), a superficial layer of cells, the enveloping layer (EVL), covers a semi-spherical cap of blastomeres centered on the animal pole of the embryo sitting on a massive yolk syncytial cell. Epiboly consists of the cortical vegetal ward expansion of the EVL, the deep cells (DCs) of the blastoderm and the external layer of the syncytial yolk cell (E-YSL) around the yolk. Epiboly ends with the closure of the EVL and the DCs at the vegetal pole (Kimmel et al., 1995; Solnica-Krezel, 2006; Rohde and Heisenberg, 2007; Figure 1A).

Epiboly progression entails a coordinated series of cellular events. EVL cells and DCs proliferate and exchange neighbors expanding and replacing the exposed yolk membrane (Betchaku and Trinkaus, 1986; Cheng et al., 2004). At the yolk cell, the E-YSL membrane becomes highly convoluted (Betchaku and Trinkaus, 1978) and gradually narrows by localized contraction (Hernandez-Vega et al., 2017). As the EVL margin advances, myosin and polymerized actin get progressively confined to a belt at the animal edge of the E-YSL and to the vegetal cap (Cheng et al., 2004; Koppen et al., 2006). In the E-YSL, actin is conscripted within and beneath the highly dynamic convoluted membrane (Hernandez-Vega et al., 2017). Actin accumulation is accompanied by myosin phosphorylation (Koppen et al., 2006). Remarkably, the narrowing of the E-YSL occurs in synchrony with cortical retrograde actin and myosin flows originating at the vegetal pole (Behrndt et al., 2012). Internally and coupled to epiboly progression, yolk granules sustain stereotyped dynamic movements (Hernandez-Vega et al., 2017).

It has been suggested that longitudinal and latitudinal tensional forces originating at the E-YSL constitute the major force-generating elements driving epiboly (Solnica-Krezel and Driever, 1994; Cheng et al., 2004; Koppen et al., 2006; Schepis et al., 2012). For the vegetal ward movement of the blastoderm some source of tension must be coupled to the contractile E-YSL. A positive vegetal ward oriented latitudinal tension gradient at the yolk cell membrane from the EVL margin could convey the stress originated by the constriction of the actomyosin ring at the E-YSL (Hernandez-Vega et al., 2017). Alternatively, or in combination, flow-friction motors could generate a pulling longitudinal force through resistance against the retrograde actomyosin cortical flows in the yolk (Behrndt et al., 2012). Yet, whatever the mechanical means involved are, epiboly must overcome the hindrance that the yolk cell membrane poses to its progression. The EVL does not slide over the yolk cell surface and is firmly attached to it (Koppen et al., 2006; Hernandez-Vega et al., 2017). Thus, as the blastoderm expands, the external yolk cell membrane subsides and it is fully eliminated (Betchaku and Trinkaus, 1986; Solnica-Krezel and Driever, 1994; Cheng et al., 2004).

To define the role of yolk cell membrane remodeling during epiboly we interfered with endocytosis by inhibiting Rab5 activity, exclusively in the yolk cell. Rab5 is a member of the Rab guanosine triphosphatases (small GTPases) family. Members of this family are essential for vesicle trafficking: Rab5 for internalization and merging into early endosomes, while other Rabs regulate exocytosis or trafficking between other organelles (reviewed in Zerial and McBride, 2001). Rab5 and the GTPase Dynamin regulate CME (Zeigerer et al., 2012) and, downstream of actomyosin contractility, act to remove membrane excess (Lee and Harland, 2010). Additionally, Rab5 has also been implicated in macropinocytosis (Tall et al., 2001; Barbieri et al., 2004; Lanzetti et al., 2004).

We first spatially and temporally characterized yolk cell membrane turnover as epiboly proceeds. Then, we found that the activity of Rab5ab, one of the four Rab5 isoforms annotated in the zebrafish, which was previously found to be important for gastrulation (Kenyon et al., 2015), is essential for yolk cell membrane turnover and epiboly movements and mechanics. In addition, we show that Rab5ab is critical for the proper recruitment of actin and myosin to the E-YSL and for their contractile activities. which affects EVL cells elongation, internal yolk flows and embryo geometry. Thus, impairing *rab5ab* expression specifically in the yolk cell alters the epiboly’s biomechanical landscape decreasing the yolk cell surface tension and leading to a reduction on the strength of the E-YSL as a mechanical power source. Altogether, our data show that localized membrane removal in the yolk cell constitutes a necessary step for epiboly progression bridging cellular, geometrical and mechanical constrains.

## Results

### E-YSL Membrane Dynamics

To study zebrafish embryo membrane turnover during epiboly we employed fluorophore-conjugated lectins. Lectins bind to glycoproteins and glycolipids and have already been used to follow plasma membrane dynamics in other teleost embryos (Cheng et al., 2004). Upon soaking the embryos in fluorophore-conjugated lectin-containing media, both, the whole yolk cell and the EVL external membranes were quickly homogeneously decorated. Immediately after, lectin-enriched spots, resembling endocytic vesicles, deposited beneath the yolk cell membrane, accumulating in a circumferential ring ahead of the EVL leading cells (Figure 1B and Supplementary Movie 1). These signs of membrane removal ahead of the EVL were observed as early as the sphere stage and co-localized with the convoluted E-YSL domain, where actin and myosin progressively gather (Behrndt et al., 2012). We observed a tight spatiotemporal correlation between the narrowing of the endocytic belt and the reduction of the width of the E-YSL (convoluted yolk cell surface; Hernandez-Vega et al., 2017; Figure 1C), which suggests an intimate relationship between both events (Cheng et al., 2004). Indeed, fluid phase endocytosis, reported by the uptake of fluorescent dextran (Feng et al., 2002; Kenyon et al., 2015), displayed the same pattern as lectin internalization (Figure 1D).

To precisely map and characterize yolk cell membrane turnover, we locally marked the membrane by laser photobleaching (employing a fluorescently labeled lectin) and followed its dynamics *in vivo*. Membrane photobleached regions away from the EVL edge remained static, potentially indicating the lack of major lateral diffusion within the yolk cell membrane, up to the time when the photobleached areas of the yolk cell got embedded by the advancing E-YSL. At this time the tagged membrane subdued and became endocytosed (Figure 1E and Supplementary Movie 2). The photobleached area linearly reduced its size and was finally eliminated before contacting the EVL margin (Figure 1F).

The observed dynamics of the yolk cell membrane confirms that the EVL does not slide over the yolk cell (Koppen et al., 2006) and suggests that its progression demands the progressive removal of the E-YSL in an animal-vegetal direction up to its full disappearance, so that the overall surface of the embryo remains constant.

### Rab5ab-Mediated Endocytosis Is Required in the Yolk Cell for Epiboly Progression

We found that the turnover of the yolk cell membrane is spatially associated with the E-YSL proximal domain. The extensive convolution of this area (Hernandez-Vega et al., 2017) points to non-clathrin dependent macropinocytosis as the mechanism for its removal.

To fully block membrane removal we set out to interfere with *rab5* expression, a key element for both vesicle internalization and targeting to early endosomes by CME and non-clathrin dependent macropinocytosis (Zerial and McBride, 2001). To target only the YSL without affecting the blastoderm, we locally injected morpholinos (MOs) into the yolk syncytium (YMOs) after the yolk cell segregation at the 512-1024 cell-stage. In this way, injected MOs remained confined to the YSL and were not mobilized to the rest of the embryo (Kimmel and Law, 1985). Yolk injection of mRNAs or fluorescently tagged MOs at these stages confirmed their restricted expression in the YSL (Supplementary Figure 1).

In zebrafish, there are five annotated *rab5* genes (*rab5aa*, *rab5ab*, *rab5b*, *rab5c* and *rab5clike*). Of these, *rab5aa*, *rab5ab*, and *rab5c* were known to be ubiquitously expressed, unlike *rab5b*, whose expression is limited to the yolk cell syncytial layer, pronephric duct, and telencephalon (Thisse et al., 2004). 1-4 cell stage MOs interferences in four of these genes (*rab5aa*, *rab5ab*, *rab5b* and *rab5c*) (Kenyon et al., 2015) indicate that while *rab5b* and *rab5c* caused no apparent effect before 24 h post fertilization (HPF), and *rab5aa* morphants are undistinguishable from control embryos, *rab5ab* morphants are embryonic lethal.

We tested whether inhibition of *rab5ab* prevented membrane removal by monitoring fluid-phase dextran endocytosis. Three different *rab5ab* morpholinos, *rab5ab* YMO1 (ATG), YMO2 (UTR) and YMO3 (UTR-ATG) injected in the yolk syncytium at the 512-1024 cell-stage caused the same phenotype, while a mismatched MO or a *rab5c* YMOs (Ulrich and Heisenberg, 2008) did not.

**FIGURE 2:**
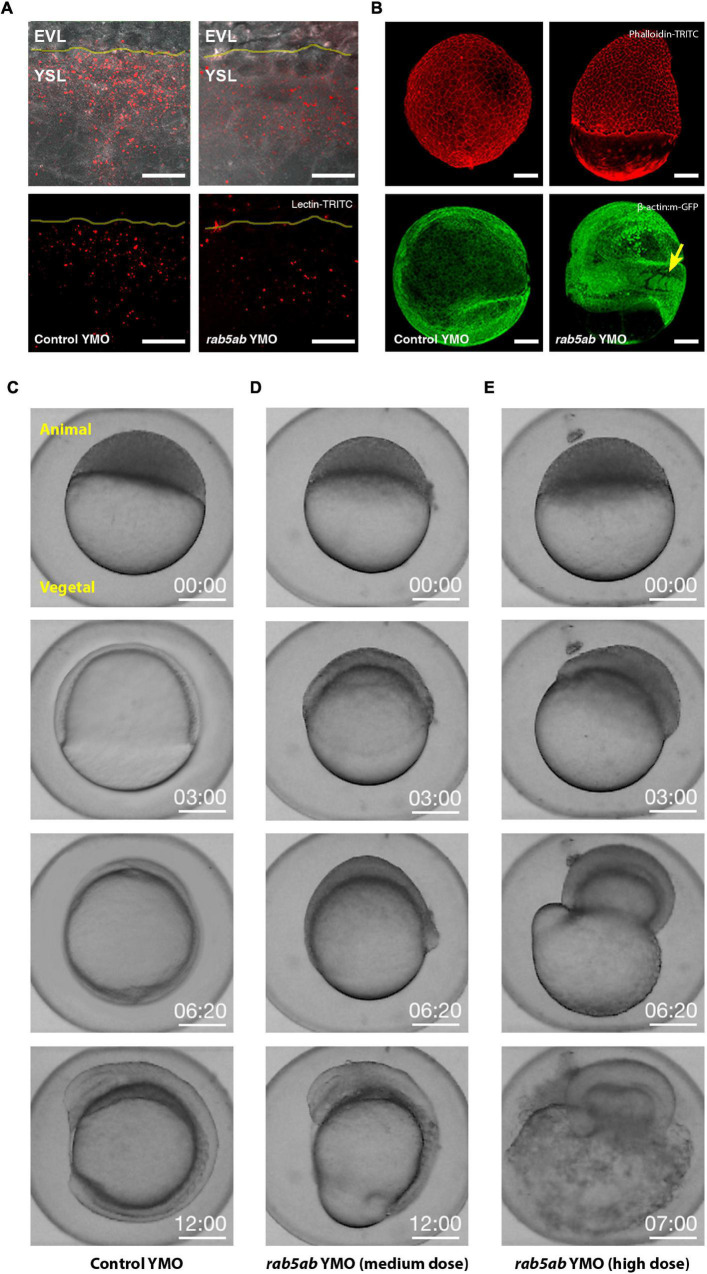
Endocytosis and Epiboly are impaired after *rab5ab* depletion. **(A)** Lectin-TRITC internalization was reduced at doming stage in *rab5ab* (right) compared to sibling controls YMOs (left). EVL and the YSL are indicated. The EVL/YSL border is highlighted. Top panels show Lectin (red) and bright field overlays. Images are confocal maximum projections. Scale bar 25 μm. **(B)** Yolk cell depletion of *rab5ab* results in epiboly delay without affecting other gastrulation movements [Control (left) and *rab5ab* YMOs (right)]. Top panels show Phalloidin-TRITC (red) staining of a control YMO at the end of epiboly and a sibling medium dose *rab5ab* YMOs of the same age. Lateral views. Bottom panels show membrane-GFP (Tg (β-*actin:m-GFP*) YMOs at 24 HPF. *rab5ab* YMOs present an open back phenotype but succeed in other gastrulation movements leading to somite formation (arrow). Images are confocal maximum projections. Scale bar 100 μm. **(C–E)** YMOs show a dose dependent epiboly delay. Macroscopic bright field images of sibling controls **(C)** and medium (4 ng) **(D)** and high (8 ng) dose **(E)** *rab5ab* YMOs (from Supplementary Movies 4, 5). Medium and high dose YMOs remained at 70 and 40% epiboly respectively when control siblings have already closed. Embryos were imaged in their chorion. The Animal and Vegetal Poles are indicated. Scale bar 250 μm.

*rab5ab* depletion just in the yolk cell led to deficient membrane removal (Figure 2A and Supplementary Movie 3) and quantitative analysis showed that the number of internalized dextran-containing vesicles in *rab5ab* YMOs was reduced by 84% (*n* = 7) when comparing with control YMOs (*n* = 12) (see section “MATERIALS AND METHODS”). *rab5ab* yolk cell specific depletion resulted in a strong early epiboly delay and arrest. Conversely, other gastrulation and morphogenetic movements (invagination, convergence and extension, and somitogenesis) and head and trunk development initiated timely and seemed mostly unaffected (see Figure 2B).

*rab5ab* YMOs displayed a dose-dependent response. Prior to epiboly no apparent defect was observed at any dosage. Phenotypes arose from dome stage onward. At a medium dose (4 ng/embryo), *rab5ab* YMOs domed in a timely manner but immediately slowed down, halting at 70% epiboly (*n* = 45). When control YMO injected sibling embryos (*n* = 62) reached the shield stage, medium dose *rab5ab* YMOs had not progressed beyond 30% and when the DCs of controls closed the yolk cell plug, *rab5ab* YMOs still remained at 60% epiboly (compare Figures 2C,D; see Supplementary Movie 4). The spherical shape of these embryos was lost and they elongate animal-wards (see Figures 2B,D). High dose yolk cell-injected embryos (8 ng/embryo) never progressed beyond 50% epiboly and burst shortly after (*n* = 36) (compare Figures 2C,E; see Supplementary Movie 5). Epiboly arrest correlated with a progressive folding of the DC layer detaching from the YSL, and with a constriction at the yolk surface ahead of the EVL margin. Alongside the epiboly delay, *rab5ab* YMOs failed to thin the blastoderm, which retracted animal ward (Supplementary Movie 5).

MO1 and MO2 had been previously employed to analyze the role of *rab5ab* in nodal signaling and gastrulation. In that context, the effect of both MOs on *gsc* expression was fully rescued by co-injection of a *rab5ab* RNA ensuring their specificity (Kenyon et al., 2015). Here we tested if the phenotype on epiboly progression observed upon *rab5ab* morpholino injection at the yolk (YMO) was reversed using RNA rescue. To ensure success, the injected rescue mRNA did not encode the MO target sequence. The construct was engineered changing the nucleotide sequence taking advantage of the degeneracy of the genetic code. These changes did not alter the encoded protein. Sibling embryos were divided into 5 experimental categories: Group 1 (*n* = 53) was injected with the *rab5ab* targeting MO (MO3); Group 2 (*n* = 47) was injected with MO3 and the synthetic *rab5ab* mRNA; Group 3 (*n* = 48) was injected with the standard control MO; Group 4 (*n* = 57) was injected with the synthetic *rab5ab* mRNA alone and Group 5 (*n* = 50) were non-injected siblings. All groups were injected at 128-256 cell stage (Supplementary Figure 2). The phenotype in each group was evaluated by bright field microscopy and calculated as percentages of normal and abnormal phenotypes. All wild type non-injected embryos or injected with a control morpholino or with *rab5ab* mRNA alone reached shield-70% epiboly and displayed a normal aspect. Instead, 75,47% of embryos injected with MO3 in the yolk were delayed and displayed the morphology described in Figures 2D,E. Rescued embryos using *rab5ab* mRNA co-injection showed morphologies, 70,17% in average, similar to those of the wild type non-injected embryos.

Altogether, these data indicate that *rab5ab* dependent endocytosis is involved in local yolk cell membrane clearance at the E-YSL and strongly suggest that its removal is necessary to maintain the mechanical equilibrium between different layers during gastrulation and the spherical shape of the embryo, and to enable epiboly progression. The absence of phenotypic defects in *rab5c* YMOs supports this conclusion, although a role for chemical signaling cannot be fully discarded.

### Rab5ab Activity in the Yolk Cell Affects Cortical Actomyosin Accumulation and, Non-autonomously, Enveloping Layer Shape and Yolk Granules Dynamics

The epiboly progression failure observed after inhibition of *rab5ab* expression in the yolk cell associated to several cellular and structural phenotypes.

**FIGURE 3:**
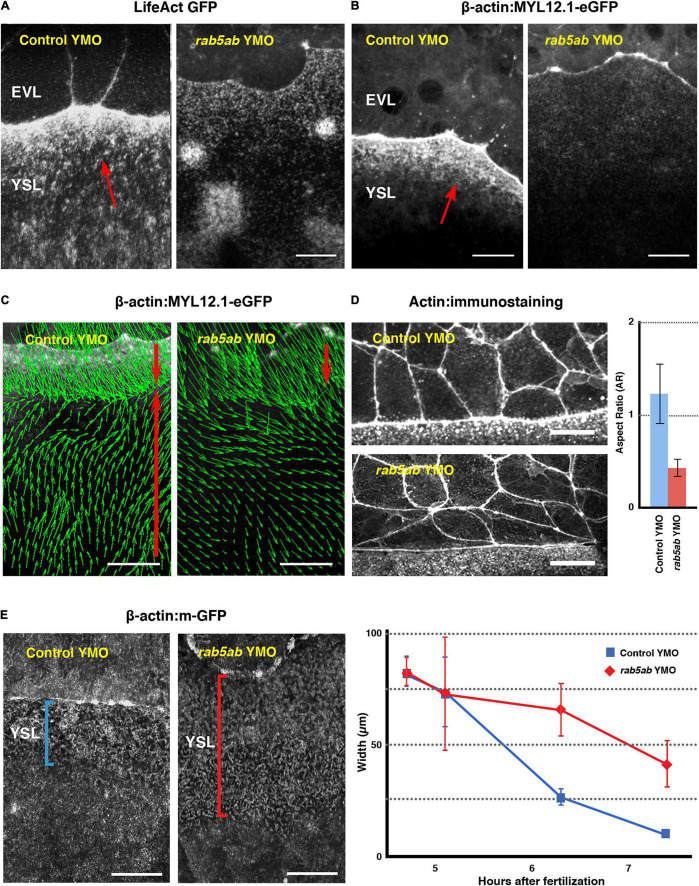
Cytoskeleton dynamics and EVL leading cells shapes are affected by *rab5ab* depletion. **(A)** Actin fails to accumulate at the E-YSL in *rab5ab* YMOs versus controls (Control YMO). Time-lapse snapshots of two LifeAct GFP injected sibling embryos. **(B)** Myosin fails to accumulate at the E-YSL of *rab5ab* YMOs versus controls (Control YMO). Time-lapse snapshots of two Myosin-GFP transgenic [Tg (β-*actin:MYL12.1-eGFP*)] sibling embryos. Note the delay in the progression of the EVL and the weaker accumulation of actin **(A)** and myosin **(B)** in the *rab5ab* YMOs. EVL and YSL are indicated. Red arrows point to the F-Actin belt and myosin accumulation in **(A)** and **(B)** respectively ahead of the EVL on the YSL. Scale bar 25 μm. **(C)** Myosin cortical retrograde flows. PIV of time-lapse snapshots of Tg (β-*actin:MYL12.1-eGFP*) embryos at 40% epiboly (from Supplementary Movie 7). Notice the vegetalward movement of cells and E-YSL (red top arrows) and the retrograde animalward cortical flow from the yolk cell vegetal pole sinking at the E-YSL (red bottom arrows), which fails in the *rab5ab* YMOs. Scale bar 25 μm. **(D)** Comparing leading EVL cells (left panels) of control siblings (Control YMO - top) and *rab5ab* YMOs (bottom) at 70% epiboly shows that they flatten and elongate latitudinally in mutant conditions. Actin was stained with phalloidin-TRITC. Scale bars 25 μm. All confocal images are maximum projections. This phenotype was quantified (on the right) by calculating the aspect ratio (animal to vegetal vs. latitudinal - Y axis) of leading EVL cells of control siblings (*n* = 15) (red) and *rab5ab* YMOs (*n* = 15) (blue) embryos. The leading EVL cells flatten at their front and elongate latitudinally in *rab5ab* morphants. Standard deviations are shown. *P*-value < 0.001. **(E)** E-YSL contraction is delayed in *rab5ab* YMOs. Snapshots at 7.5 HPF from surface projections of membrane-GFP (Tg (β-*actin:m-GFP*) *rab5ab* and sibling YMOs (Left Panels). Between 4.5 to 7.5 HPF, the E-YSL width is reduced from 80 to 10 μm as an average in controls (*n* = 5) (blue), but from 80 to 40 μm in *rab5ab* YMOs (*n* = 5) (red). Standard Deviations are shown. *P*-value < 0.001 from 6 h onward.

**FIGURE 4:**
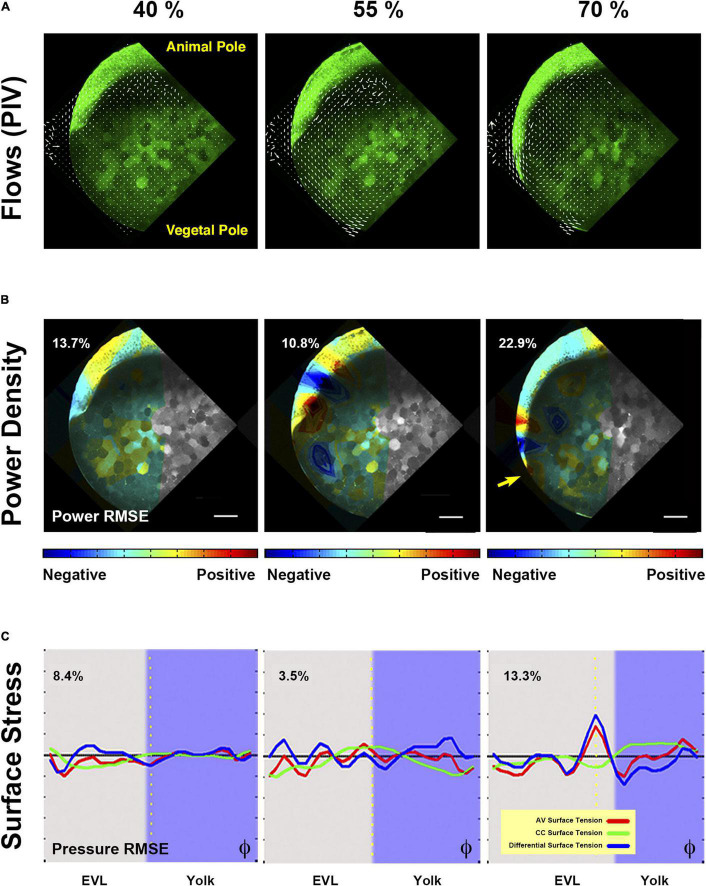
Biomechanics of yolk cell endocytosis impaired embryos. **(A)** Yolk granules flows patterns are altered in *rab5ab* YMOs. PIV of time-lapse snapshots imaged by two-photon microscopy of a Tg (β-*actin:m-GFP*) *rab5ab* YMO (see Supplementary Movies 8, 9). From epiboly onset, yolk granules flows are uncoordinated in *rab5ab* YMOs. The internal toroidal vortices characteristic of epiboly progression (Hernandez-Vega et al., 2017) do not form or are severely reduced and the laminar flows become asymmetrically distributed. Animal (A) and vegetal (V) poles are indicated. **(B)** Mechanical power density maps over time obtained by HR analysis in *rab5ab* YMO (from Supplementary Movie 10). The Relative Mean Square Error (RMSE) of the inferred power maps is shown as a percentage at the top left of each panel expressing the fitting accuracy. Qualitatively, *rab5ab* YMOs display no differences with wild type embryos in the spatial distribution of mechanical work (Hernandez-Vega et al., 2017) but exhibit a strong delay and an overall decrease of power of about four-fold. Arrow points to the highest power value at 70% epiboly at the E-YSL. Scale bar 25 μm. **(C)** Longitudinal along the AV axis (red) and latitudinal or circumferential - CC (green) stresses and their differences (blue) along the embryo cortex in a membrane-GFP transgenic [Tg (β-*actin:m-GFP*)] *rab5ab* YMO (from Supplementary Movie 12). Stresses were plotted as a function of the φ angle from animal to vegetal (40, 50, and 65% epiboly). The equator - dotted yellow line -, yolk cell surface - purple shadow - and the RMSE of the dynamic pressure (fitting accuracy) as a percentage for each time point are displayed. The latitudinal stress does not steep up from animal to vegetal until 70% epiboly, while the longitudinal stress oscillations are sustained.

(1) The local recruitment of actin and myosin to the E-YSL was compromised by middle dose interference in *rab5ab* expression in the yolk cell. The levels of both proteins, detected with LifeAct-GFP and Myosin-GFP respectively, were strongly reduced in *rab5ab* YMOs (Figures 3A,B). These reductions most probably relate to defects in the retrograde cortical myosin flow observed in wild type animals (Behrndt et al., 2012). Live time-lapse imaging of transgenic Tg (β-*actin:MYL12.1-eGFP*) embryos and Particle Image Velocimetry (PIV) analyses (see Materials and Methods) revealed that the magnitude of the yolk cell cortical myosin flows decreased and that their directionality is altered in *rab5ab* YMOs when comparing with control YMOs (Figure 3C and Supplementary Movie 6).
(2) The autonomous effects on the yolk cell cortical actomyosin dynamics were accompanied by non-autonomous changes in the shape of EVL cells. In wild type embryos actin accumulation at the E-YSL has been correlated with EVL cells elongation in the animal-vegetal (AV) direction at the margin (Koppen et al., 2006). We found this was prevented in *rab5ab* YMOs, where the leading EVL cells elongated in the dorsal to ventral (DV) direction (Figure 3D and Supplementary Movie 7). We reasoned that these altered shapes respond to changes in the tension anisotropy within the E-YSL, which increases in normal conditions, as a rule, as epiboly progresses (Hernandez-Vega et al., 2017).
(3) The narrowing of the actin-rich convoluted E-YSL, the major source of force generation during epiboly (Hernandez-Vega et al., 2017), was also affected. The E-YSL narrowed at a slow pace and never shrink in full (the width of the E-YSL was quantified from surface projections of membrane-GFP tagged control and rab5ab YMO injected embryos (transgenic Tg(β*-actin:m-GFP*) of different ages – see section “MATERIALS AND METHODS”) (Figure 3E).
(4) Finally, we found that the stereotyped movements of yolk granules, which are known to passively respond to the cortical stresses created at the E-YSL (Hernandez-Vega et al., 2017), became disturbed upon *rab5ab* depletion. Velocity fields, estimated by PIV from meridional multiphoton microscopy sections, showed that the yolk granules regular toroidal vortices associated with epiboly (Hernandez-Vega et al., 2017) were severely affected (see Figure 4A and Supplementary Movie 8). They became uncoordinated showing a noticeably slower kinetics.

In summary, our data indicate that membrane removal at the E-YSL mediated by Rab5ab, is necessary for the correct structural organization and activity of the E-YSL. Endocytic activity in the yolk cell, ahead of the EVL, most probably influence E-YSL contractility, non-autonomously affecting in turn both EVL cells elongation and the pattern of yolk granules flows.

### Epiboly Mechanics in *rab5ab* YMOs

We found that in addition to structural and functional defects of the E-YSL and epiboly arrest, the overall geometry of *rab5ab* YMOs was affected. The final shape of the embryos became rather an ellipsoid than a sphere with an elongated animal to vegetal (AV) axis (see Figure 2 and Supplementary Movie 4). This elongated shape resulted from both the animal ward expansion of the blastoderm after 50% epiboly and the slight elongation of the yolk cell toward the vegetal pole. The altered geometry of these embryos suggests that their global mechanical balance was compromised and that the spatial distribution and dynamics of stresses during epiboly were disrupted. To explore this possibility, we analyzed the spatio-temporal profile of mechanical power and cortical tension of *rab5ab* YMOs by Hydrodynamic Regression (HR) (Hernandez-Vega et al., 2017). Briefly, in wild type embryos HR reveals a stereotyped mechanical power density pattern throughout epiboly. At its onset, most mechanical activity maps to the blastoderm. Then, once the EVL crosses the equator, the largest mechanical power density is found in the active, actomyosin-rich, E-YSL while the adjacent EVL cells oppose deformation. As a consequence, a gradient of tension pointing toward the vegetal pole progressively develops at the yolk cell surface (Hernandez-Vega et al., 2017).

To define the biomechanical make up of *rab5ab* YMOs we employed experimental 2D velocity fields obtained by PIV from time-lapse imaging of meridional sections with the yolk granules movements as a reference (Figure 4A, Supplementary Figure 3 and Supplementary Movie 9). These analyses showed that the yolk flows of *rab5ab* YMOs (*n* = 6) were severely impaired versus control YMO (*n* = 5).

The velocity fields were simulated on a spherical cortex model and fitted by HR (Hernandez-Vega et al., 2017). This let to infer mechanical power densities and surface tension maps.

Power density data is represented in heated 2D maps overlaying the meridional sections (Figure 4B and Supplementary Movie 10). Red represents the largest mechanical power (positive values) and blue elastic resistance to deformation (negative values). Through this analysis, we found that upon interference in *rab5ab* expression in the yolk cortex, the overall mechanical energy detected at the yolk cortex was strongly reduced when compared to control YMO embryos. Yet, its spatial and temporal distribution with maximum levels at the actomyosin-rich YSL by 70% epiboly was very similar to that of wild type embryos (Supplementary Figure 4 and Supplementary Movie 11). See also (Hernandez-Vega et al., 2017).

Surface tension maps for animal to vegetal (AV) and circumferential (CC) cortical stresses as well as differential surface tension inferred by HR were represented by linear graphs along the AV axis relative to the advance of the EVL front. This type of representation lets distinguish differences in stress along the axis as a function of time. We found that at the initiation of epiboly the AV and CC stresses are evenly distributed at the surface of the embryo, both in *rab5ab* and control YMOs. As epiboly progresses, the longitudinal (AV) and latitudinal (CC) surface stresses get weaker in rab5ab YMOs. The latitudinal stress gradient in rab5ab YMOs develops only at late stages (Figure 4C, Supplementary Figure 5 and Supplementary Movie 12).

In summary, Rab5ab-mediated yolk cell endocytosis does not influence where mechanical power builds up during epiboly but is necessary to reach a proper level of cortical tension at the right time.

**FIGURE 5:**
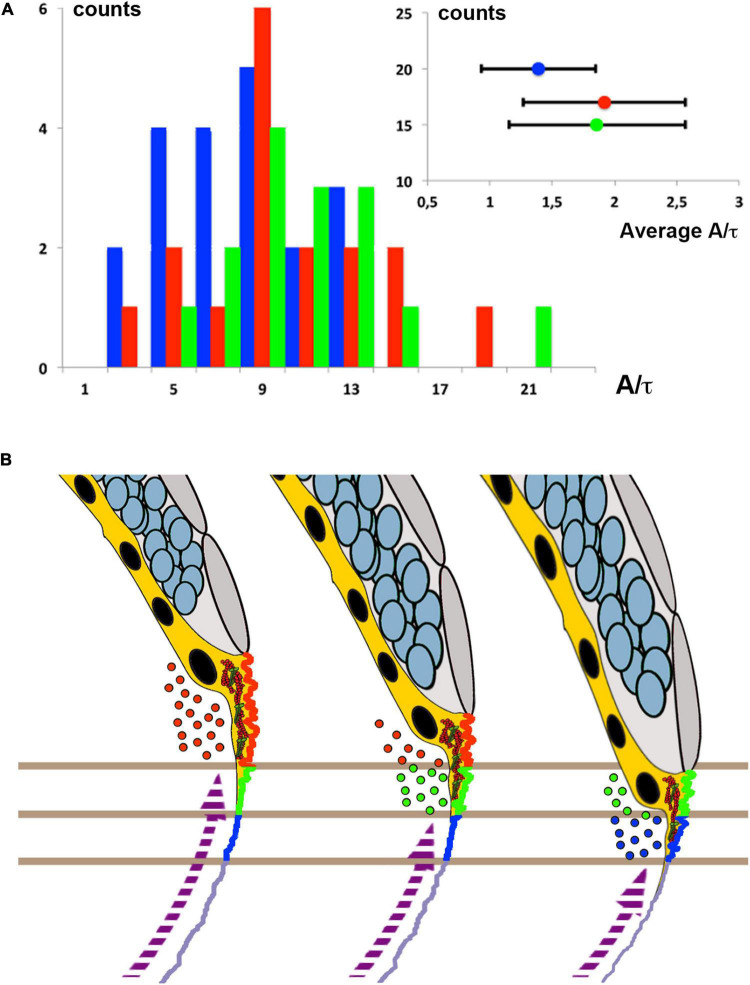
Membrane cortical tension and endocytosis at the E-YSL are necessary for epiboly progression. **(A)** The distribution (counts) of instant retraction velocities (A/t) after laser surgery of the actomyosin cortex of Myosin-GFP (Tg (β-*actin:MYL12.1-eGFP*) *rab5ab* YMOs at 55% epiboly (blue) shows a significant reduction (Wilcoxon test *p* < 0.01) versus wild type (red) and control YMOs (green). The instant velocity estimate was extracted from the exponential fit of the distance between fronts (see section “MATERIALS AND METHODS”). The averaged instant retraction velocity (A/t) for *rab5ab* YMOs was 1.39 ± 0.46 μm (*n* = 20), while control YMOs reach 1.86 ± 0.71 μm (*n* = 17) and wild type embryos 1.92 ± 0.65 μm (*n* = 15) (inset). Counts represent the number of analyzed laser cuts for each condition. **(B)** Proposed model of epiboly progression. The contractile E-YSL and the imbalance of stiffness between the EVL and the yolk cell surface account for epiboly progression. Rab5ab-mediated yolk cell localized endocytosis (color coded dots) accounts for the reduction of the yolk cell surface coupled to the progression of EVL (gray) and DCs (blue) toward the vegetal pole. Membrane removal associates to the convolution and contraction of the E-YSL surface and the recruitment of actin and myosin (purple dashed arrow) from vegetally located pools. Three chronological time points are shown. Different sequential zones on the surface of the E-YSL are color-coded. Actin and myosin are diagrammatically illustrated in red and green within the YSL (yellow).

To corroborate the differential tensional topology of the yolk cell cortex inferred by HR we employed laser microsurgery (Colombelli et al., 2009). It has been shown that incisional cuts of the cell cortex result in its immediate recoil with an exponentially decaying speed proportional to its tensional level before ablation (Grill, 2011) (Hernandez-Vega et al., 2017). Employing this approach we found that blocking membrane removal led to a reduction of surface tension (Figure 5A, Supplementary Figure 5A). Further, laser cuts performed parallel to the EVL at different distances from the margin, indicate that the gradient of tension along the A/V axis generated on the yolk surface [1.57 ± 0,46 times at a distance of 60 μm of the EVL edge (*n* = 11) with respect to a 20 μm reference (*n* = 17) in control YMO] does not develop by 65% epiboly (*p* < 0,05) in *rab5ab* YMOs [1,04 ± 0.44 times at 60 μm (*n* = 8) with respect to the 20 μm reference (*n* = 21)] (Supplementary Figures 5B,C). This is in agreement with the steady surface tension inferred by HR along the AV axis for *rab5ab* YMOs (Figure 4C). Summing up, the vegetal ward gradient of tension on the yolk cell associated to epiboly progression (Hernandez-Vega et al., 2017) is delayed and weakened in *rab5ab* YMOs.

All in all, our data indicate that local membrane removal is essential for strengthening latitudinal (CC) and longitudinal (AV) forces at the E-YSL and for the development of an anisotropic gradient of tension at the cortex. These properties, jointly, would govern epiboly movements.

## Discussion

The function of plasma membrane remodeling during morphogenesis is recently receiving high attention. Indeed, the balance between endocytosis and recycling seems to be critical to regulate cell morphology and tissue deformations in multiple morphogenetic processes. Rab5-mediated endocytosis is required downstream of acto-myosin contraction to remove excess membrane in bottle cells in *Xenopus* and to promote their coordinated constriction (Lee and Harland, 2010). Likewise, Rab5 is also required in the amnioserosa during dorsal closure in *Drosophila* for membrane removal as cells delaminate (Mateus et al., 2011). In zebrafish, different Rab5 isoforms appear to have different roles, participating in Nodal signaling in early embryos affecting the development of the dorsal organizer (Rab5ab), or muscle and brain development (Rab5b and Rab5c respectively) (Kenyon et al., 2015). On the other hand, during cellularization in fly embryos, Rab11, which mediates vesicle recycling, seems to regulate membrane growth and invagination and the elongation of epidermal cells (Pelissier et al., 2003).

The contractile capabilities and gradual change of dimensions of the E-YSL together with the distinct elastic properties of the EVL and the yolk cell surface minimally account for epiboly kinematics and mechanical behavior from 50% epiboly onward (Hernandez-Vega et al., 2017). The E-YSL exerts an isotropic contractile force that generates stress at the adjacent EVL at its animal side and the more vegetal yolk cell cortex. These have different mechanical properties. The EVL is easily deformed by this pulling force and passively expands changing the shape of its cells. On the opposite, the thin yolk cell cortex cannot stretch in response to contraction. In this scenario, we propose that localized membrane removal at the E-YSL is essential for the effective movement of the EVL toward the vegetal pole (Figure 5B). The non-convoluted yolk cytoplasmic layer would be progressively recruited to the adjacent E-YSL as this is eliminated. Throughout epiboly, to keep the yolk cell surface balanced, the membrane removal at the front of the advancing EVL must be compensated by membrane recruitment at the I-YSL. Membrane turnover compensation (exocytosis) in the yolk cell remains to be explored. Yet, the coupling of exocytosis and endocytosis observed in EVL cells during epiboly (Ahn et al., 2010) gives evidences for such potential outcome.

Endocytosis of the E-YSL surface was previously suggested to contribute to epiboly (Betchaku and Trinkaus, 1986; Solnica-Krezel and Driever, 1994). However, knock-down of Dynamin 2 in the yolk cell had little effect on epiboly progression (Lepage et al., 2014) suggesting that the endocytic removal of the yolk cell membrane was dispensable. Yet, Dynamin 2 dependent membrane endocytosis only accounts for CME. We found that depletion of *rab5ab* leads to epiboly arrest and was very efficient in preventing yolk cell membrane removal. These results point to macropinocytosis and not to CME as the main mechanism involved in membrane trafficking in the E-YSL. Macropinocytosis is characterized by large non-selective membrane internalization and by the presence of actin cytoskeleton protrusions (ruffles) and has been previously proposed as a plausible mean for membrane remodeling (Swanson and Watts, 1995; Cao et al., 2007; Lim and Gleeson, 2011). Indeed, forced macropinocytosis elicited by injection of human Rab5a mRNA in one-cell stage embryos robustly accelerates epiboly progress (Malinverno et al., 2017). While, in principle, the epiboly defects observed by reduction of *rab5ab* expression in the YSL could be connected to pleiotropic cell signaling faults, their absence upon interference of other Rab5 isoforms in the yolk cell [e.g., Rab5c (Ulrich and Heisenberg, 2008; Song et al., 2013)] underline their potential link to bulk effect on membrane removal.

A common feature of endocytic membranes, as opposed to other passive regions of the plasma membrane, is their high curvature (Doherty and McMahon, 2009). This curvature is somehow linked to the presence of a specific set of regulatory proteins, many of them necessary for curvature generation (Kozlov et al., 2014). Further, endocytic membrane curvature also appears to be influenced by cytoskeleton motor proteins such as myosin (Spudich et al., 2007). In addition to pushing forces mediated by actin polymerization (helping, e.g., to push neck membranes closer together), the cytoskeleton may also provide pulling forces to keep vesicle necks under tension (Roux et al., 2006). Our data indicate that actomyosin contractility may be necessary at the E-YSL for membrane folding and be required to fold the E-YSL membrane into ripples.

Endocytosis of the E-YSL appears to be key for proper epiboly progression, we found that in the absence of Rab5ab, (1) the overall power and the longitudinal and latitudinal stresses (and the shear stress) at the cortex were severely disturbed, and (2) the gradient of tension along the φ axis of the yolk cell surface was weakened (Figure 4C). We also found that the yolk cell cortical tension suffers a significant reduction (Figure 5A) probably associated to a decrease on the levels of actin and myosin in the E-YSL cortex (Figures 3A,B). This decline most probably affects E-YSL cortex contractile capability. In summary, during epiboly, initial E-YSL actomyosin contractility is followed by yolk cell membrane endocytosis, which seems to further potentiate localized cortex constriction implementing a positive mechanical loop.

The mechanical unbalance consequence of the failure in yolk cell membrane removal after interference in *rab5ab* expression results in a loss of the embryo sphericity, which is linked to the epiboly arrest. Further, both, epiboly arrest and an anisotropic embryo shape have been observed following whole embryo knockdown of Ap2a1, the main adaptor molecule for CME (Umasankar et al., 2012). Yet, knockdown of other genes, *a priori* unrelated to membrane removal, also results in equivalent morphogenetic defects (Pei et al., 2007; Ahn et al., 2010) suggesting that embryo elongation is more a consequence of the unability of the EVL to expand vegetal-wards around the yolk cell, than to the yolk cell membrane removal *per se*.

Mounting evidence points to a direct relation between membrane reservoir and trafficking pathways with tension in the regulation of cell shape changes and movements in morphogenetic processes (Dai and Sheetz, 1995; Sheetz and Dai, 1996; Dai et al., 1998; Apodaca, 2002; Gauthier et al., 2011, 2012; Kremnyov et al., 2012; Diz-Munoz et al., 2013). During morphogenesis, as tissues change their shapes and sizes, cell membranes dynamically change their area, composition and links to the cortex. As a consequence, membrane tension is subjected to constant modulation (Clark et al., 2014; Figard and Sokac, 2014). How membrane tension is integrated with the cell’s overall mechanical properties is unknown. In teleosts, pioneering studies in loach uncovered a direct correlation between surface membrane folds and endocytic-rich domains in early eggs. Further, the experimental decrease of loach eggs surface tension by volume reduction was found to lead to a tightly packed folding of their membrane (Ivanenkov et al., 1990). Alongside, in *Fundulus heteroclitus* embryos, mechanical deformations affect epithelial apical membrane turnover (Fink and Cooper, 1996). Yet, these early studies failed to provide a comprehensive view of the links between membrane removal, tension and morphogenetic movements.

We propose that, in the early zebrafish embryo, the surface membrane tension constitutes a mechanical buffering system constantly maintained by endocytosis and contractile activity at the E-YSL that regulates epiboly progression. The rates of removal of E-YSL membrane would vary with time and would be proportional to the tension of the yolk cell surface. Endocytosis will lead to membrane tension anisotropies in the yolk cell surface and these will mechanically feedback to regulate membrane dynamics. This mechanical loop alongside the concerted actions of latitudinal and longitudinal forces at the E-YSL would direct epiboly movements.

It has recently been reported that Rab5 controls a diverse set of collective movements by promoting directional locomotion. In this scenario, multicellular cohorts change their mechanical properties in response to membrane trafficking (Malinverno et al., 2017). Mechanical loops set up by membrane remodeling could constitute a common way to coordinate tissue movements in morphogenetic processes.

## Materials and Methods

### Zebrafish Lines Maintenance

AB and TL wild type strains were used throughout this study. Membrane-GFP transgenic [Tg (β-*actin:m-GFP*)] fish (Cooper et al., 2005) were provided by Lilianna Solnica-Krezel and Myosin-GFP transgenics [Tg (β-*actin:MYL12.1-eGFP*)] (Behrndt et al., 2012) by Carl-Philipp Heisenberg. Adult fish were cultured under standard conditions and staged embryos were maintained at 28.5°C in embryo medium (Westerfield, 2000).

### mRNA and Morpholino Injections

A DNA construct encoding for LifeAct-GFP (Riedl et al., 2008) and cloned in a Zebrafish expression vector was provided by Erez Raz. mRNA was *in vitro* synthesized (μMessage Machine kit, Ambion) and injected into the yolk at one- or 512-cell stages (150 pg). To knockdown *rab5ab*, morpholino yolk injections (4 ng and 8 ng) were performed at the 512-1024-cell stage.

### Morpholinos

Morpholinos (MOs) were purchased from Gene Tools and designed against selected regions (ATG or UTR) of the *rab5ab* gene (Accession Number ENSDARG00000007257): MO1-ATG (5-TCGTTGCTCCACCT-CTTCCTGCCAT-3), MO2-UTR-ATG (5-ACCTCTTCCTGCCATGACCCAAAAC-3), MO3-UTR (5-GACCCAAAACCCCAATCTCCTGTAC-3) and a mismatch MO (5-TCcTTcCTCgACCTCTTCgTcCCAT-3) (mispaired nucleotides in lower case). Interference with *rab5c* (ENSDARG00000026712)] was performed with the following MO: 5-CGCTGGTC-CACCTCGCCCCGCCATG-3 provided by C.P. Heisenberg (Ulrich and Heisenberg, 2008). For all experiments, a group of embryos was injected with a Standard Control MO (5-CCTCTTACCTCAGTTACAATTTATA-3).

### *In vitro* mRNA Synthesis for Rescue Experiments

The full-length coding sequence of *rab5ab* was PCR amplified and cloned into the pCS2+ vector. The digested product was then ligated and transformed into Dam- DH5α competent E. coli cells (Takara, Japan). The recombinant *rab5ab*-pCS2+ plasmid was linearized using the *Xba*I restriction enzyme. Then, mRNA was transcribed using an mMESSAGE μMACHINE T3 Transcription kit (Ambion, CA, United States). A mixture of 4 μg/μl YMO3 (UTR) and synthesized *rab5ab* mRNA (90ng/μl) was used for rescue experiments.

### Actin, Myosin and Nuclear Staining

Zebrafish embryos were fixed overnight in 4% paraformaldehyde at 4°C, washed in 0.3% Triton in PBS (PBT) and manually dechorionated. They were then washed in PBT, followed by a 2-h incubation in blocking solution (1% bovine serum albumin in PBT). Embryos were then incubated either for 1-h in blocking solution containing 0.2 μg/μl Phalloidin-TRITC (Molecular Probes, Invitrogen) at room temperature. DAPI was used for nuclear counterstaining. After incubation, embryos were washed 4 times for 15 min in PBT. For imaging, embryos were mounted on dishes with 0.5% low melting agarose (A9045 Sigma) in PBS medium. Images were acquired on a Zeiss LSM700 confocal microscope with 10 X/0.3 and 63 X/1.40 oil objectives.

### Live Imaging and Analysis

Whole embryo images were collected from non-dechorionated animals aligned in a 1.2% agarose mold and covered by E3 medium. Images were acquired (4X magnification) every 5 minutes with an Olympus MVX10 Macroscope.

For confocal and spinning-disk microscopy, embryos were mounted in 0.5% low melting agarose (A9045 Sigma) in E3 embryo medium.

Sagital sections (350 μm depth from the yolk cell membrane surface) were collected from [Tg (β-*actin: m-GFP*)] embryos using a Leica SP5 two-photon microscope equipped with a mode-locked near-infrared MAITAI Laser (Spectra-Physics) tuned at 900 nm, with non-descanned detectors and with a 25 X/0.95 water-dipping objective. Images were scanned at 200 Hz and frames were averaged three times. Stacks of 30 μm, 10 μm step-size, were acquired every 2 min.

Dextran and lectin internalization were monitored from dechorionated embryos previously incubated in 0.05% 10.000 MW Rhodamine B-Dextran (Life Technologies) for 10 min (Feng et al., 2002) or 100 μg/ml lectin-TRITC (Sigma L1261) for 5 min at the sphere stage, both diluted in E3 embryo medium. The Lectin-TRITC used was from *Helix pomatia* (Fink and Cooper, 1996), which binds *N*-acetyl-D-galactosamine and *N*-acetyl-D-glucosamine residues of glycoproteins and glycolipids on the cell surface. After treatment, embryos were rinsed in E3 medium, mounted in 0.5% low melting agarose and imaged in a Zeiss LSM700 confocal microscope with a 40 X/1.3 oil immersion objective. A stack of 20 μm, 0.39 μm step size, was acquired every 4 min.

To visualize myosin cortical flows, spinning-disk images were captured from [Tg (β-*actin:MYL12.1-eGFP*)] embryos on either an Olympus X81 inverted microscope (Andor Technologies), using a 40 X/0.60 Dry objective or a Zeiss Axiovert 200M inverted microscope (PerkinElmer UltraView ERS) using a 40 X/1.3 oil DIC objective. Stacks of 16 μm, step size 1 μm, were acquired every 45 seconds.

To visualize the surface of the yolk cell, [Tg (β-*actin: m-GFP*)] embryos were imaged in a Zeiss LSM700 confocal microscope with a 63 X/1.4 oil objective. A stack of 25 μm, step size of 0.2 μm was acquired. We also used embryos injected with *LifeAct-GFP* at the 512-cell stage and collected the images with a Zeiss Axiovert 200M inverted microscope (PerkinElmer UltraView ERS) using a 100 X/1.4 oil DIC objective. Stacks of 10 μm, step size of 0.45 μm, were acquired every 12 s.

For photo-bleaching, selected ROIs were created for lectin-TRITC soaked embryos and bleached using 100% power of a 555 nm laser with 100 iterations in the selected area (in the YSL at 150 μm from the EVL margin) in embryos at 40% epiboly. A stack of 4 μm, step size 1 μm, was acquired every 30 seconds.

Most image analyses and processing were performed using Fiji^1^ and Matlab (Mathworks). To measure the width of the wrinkled area, surface projections at different stages were obtained with Fiji and mean width and standard deviations were plotted (Excel, MS Office). To quantify endocytosis, E-YSL dextran-containing vesicles were monitored from maximum projections of Z-stack images. To obtain velocity fields we applied the MatPIV software package written by Johan Kristian Sveen for use with Matlab (Supatto et al., 2005).

### Hydrodynamics Regression

Hydrodynamics Regression (HR) is based in fitting analytically modeled velocity fields to experimental velocity fields in and outside a cortex. Considering that in deforming tissues, stresses at the fluid/cortex boundary are continuous (boundary condition), HR can estimate cortical stresses and retrieve the complete dynamic pressure distribution in the fluid and at the fluid-cortex interface. From these, HR also infers at each time point the cortex shear stress at each point of the surface and the mechanical power density. HR is performed independently at each time point to retrieve the overall spatio-temporal distribution of all these mechanical quantities (Hernandez-Vega et al., 2017).

In our analyses, experimental 2D velocity fields were estimated by PIV from time-lapse imaging of meridional sections of zebrafish embryos. Second, simulated 3D velocity fields generated from a spherical cortex model (SC) with Stokeslets pairs distributed on a single spherical shell were fitted to the experimental velocity fields. Last, knowing the fluid deformation rates, it is possible to calculate the local values of the cortical surface tension, the cortical mechanical power density and the spatio-temporal evolution of both cortical stresses and mechanical power density maps (analytical codes are available on (Hernandez-Vega et al., 2017).

### Laser Surgery Experiments and Retraction Analysis

Laser surgery of the actomyosin cortex was performed with a pulsed UV laser (355 nm, 470 ps per pulse) by inducing plasma-mediated ablation as described before (Colombelli et al., 2009). To compare the cortical tension in the longitudinal direction at the E-YSL a 20 μm-laser line containing 50 pulses was scanned 5 times at a frequency of 500 Hz, parallel to the EVL front, centering the cut at a distance of about 20 μm, through a 63 X/1.2 W objective lens. Fluorescence imaging was performed through a custom spinning Nipkow disk unit equipped with a 488 nm laser line and a Hamamatsu ORCA CCD camera, acquiring at 1.5 frames per second. Transmission and fluorescence imaging was performed by alternated illumination with two out-of-phase mechanical shutters blocking the 488 nm laser and the halogen bright field lamp.

We followed the accepted assumption (Colombelli et al., 2009) that the tension present in the actomyosin cortex before the laser cut is proportional to the outward velocity of the immediate recoil. Retraction analysis was performed through a customized kymograph analysis along the retraction axis (perpendicular to the cut), with Fiji^1^. Kymograph processing included subtraction of the intensity minimum and normalization to the maximum, both measured in the position of the cut, to ensure stable edge detection by intensity threshold across the whole sequence. The front-to-front length, during the retraction phase (until reaching a plateau), was fitted to an exponential function (Igor Pro 6.0, Wavemetrics) to evaluate the slope at the origin.

The function used was:


F(t)=y0+A[1-exp(-t/τ)]

and the slope at the origin was derived from its derivative:


dF(0)/dt=A/τ

The width of photo bleaching (about 1 μm) introduced by the UV laser was subtracted to the measured length L. This method was applied to the comparative analysis of YMOs conditions.

## Data Availability Statement

The original contributions presented in the study are included in the article/Supplementary Material, further inquiries can be directed to the corresponding author.

## Ethics Statement

The animal study was reviewed and approved by CEEA-PCB - Institutional Animal Care and Use Committee Barcelona Science Park.

## Author Contributions

MM and AH-V performed all biological tests and contributed equally to the study. P-AP developed the modeling and the regression analysis. EM-B designed the study, analyzed the data, and wrote the manuscript. All authors discussed the results and commented on the manuscript. All authors contributed to the article and approved the submitted version.

## Conflict of Interest

The authors declare that the research was conducted in the absence of any commercial or financial relationships that could be construed as a potential conflict of interest.

## Publisher’s Note

All claims expressed in this article are solely those of the authors and do not necessarily represent those of their affiliated organizations, or those of the publisher, the editors and the reviewers. Any product that may be evaluated in this article, or claim that may be made by its manufacturer, is not guaranteed or endorsed by the publisher.

## Footnotes

1
https://imagej.net/software/fiji/

## Supplementary Material

Supplementary Figure 1 Yolk Cell-Specific Morphants (YMOs). Fluorescent image of an embryo injected with a fluorescently labeled Morpholino (FITC) at the 512 cells stage and observed at 40% Epiboly. The Animal and Vegetal Pole are indicated. The red arrow points to the fluorescence in the yolk cell, accumulating at the E-YSL. Scale bar 200 μm. The inset shows at high magnification the area in which the labeled morpholino was incorporated (E-YSL) adjacent to the EVL (delimited by a green dotted line). Arrows point to the YSL nuclei which do not incorporate the injected morpholino. Scale bar 25 μm.

Supplementary Figure 2 *rab5ab* YMO rescue. The phenotype of *rab5ab* YMO embryos (MO3) (*n* = 53), embryos injected with *rab5ab* mRNA alone (*n* = 48), *rab5ab* YMO + *rab5ab* mRNA injected embryos (*n* = 57) and standard Control YMO embryos (*n* = 47) were evaluated by comparison to wild-type non-injected embryos (*n* = 50) at shield - 70% epiboly stage. The result is represented as the percentage of normal (blue bar) and abnormal (red bar) embryos in each group.

Supplementary Figure 3 Yolk granules flows patterns in *rab5ab* YMOs. PIV of time-lapse snapshots imaged by two-photon microscopy Control and *rab5ab* YMO (medium and high dose) embryos (from Supplementary Movie 9). Meridional sections (350 μm depth from the yolk cell membrane surface) were collected from [Tg (β-*actin: m-GFP*)] embryos. While Control embryos undergo epiboly on time, medium dose *rab5ab* YMOs delay and their yolk granules flow patterns are altered. High dose *rab5ab* YMOs eventually burst. Scale bar 200 μm. Time in hours.

Supplementary Figure 4 Comparison of the mechanical properties of Control and *rab5ab* YMOs. **(A–C)** Snapshots of sequential times of Control and medium dose *rab5ab* YMOs at different times of development (from Supplementary Movie 11). Mechanical power density maps (discrete power level tracks representation) obtained by HR analysis over time. Qualitatively, *rab5ab* YMOs display no differences with Control YMOs in the spatial distribution of mechanical work at early times and at 55% epiboly (red arrows). They also reach a final equivalent distribution although at weaker levels (yellow arrows) after 70% epiboly. *rab5ab* YMOs take much longer to reach this developmental stage than Control YMOs Red shaded timing. Times are in hours. Scale bar 100 μm. **(D)** and **(E)** Longitudinal (red) and latitudinal (green) stresses and their differences (blue) along the embryo cortex at two different times (55% and 75% epiboly) in membrane-GFP transgenic [Tg (β-*actin:m-GFP*)] Control and *rab5ab* YMO as in Figure 4. The equator - dotted yellow line - and yolk cell surface - purple shadow - are displayed. The latitudinal stress does not steep up from animal to vegetal in *rab5ab* YMO until 70% epiboly at a much later time that Control YMOs. Times are in hours.

Supplementary Figure 5 Animal/Vegetal gradient of Tension: laser microsurgery. **(A)** Laser surgery of the actomyosin cortex was performed with a pulsed UV laser (355 nm, 470 ps per pulse) by inducing plasma-mediated ablation. To compare the cortical tension in the AV directions, a 20 μm laser line containing 50 pulses was scanned five times at a frequency of 800 Hz, parallel the EVL front, centering the cut at a distance of about 20 μm. Scale bar 10 μm. **(B)** This laser ablation regime (same laser line length) was employed at 20 and 60 μm to study the surface gradient of tension in *rab5ab* and Control YMOs at 65% epiboly). Scale bar 10 μm. **(C)** To quantify the AV surface gradient of tension, we normalized the recoil velocities at each distance from the EVL margin to the recoil velocity at 20 μm distance and tested the statistical significance of the gradient using the Wilcoxon rank sum test. The gradient does not develop in *rab5ab* YMOs (*P* < 0.05) when comparing to Control YMOs.

Supplementary Movie 1 Endocytosis. Confocal video time-lapse of a wild type animal soaked in lectin–TRITC for 5 min at sphere stage. 20 μm width stacks were captured every 4 min. Red arrow points to E-YSL. Note the cumulative lectin-TRITC internalization at the E-YSL domain ahead of the advancing EVL.

Supplementary Movie 2 Endocytosis at the E-YSL. HiLo LUT code (Fiji) processed confocal video time-lapse of a lectin-TRITC soaked embryo showing a circular photo bleached ROI in the yolk cell 60 μm away from the EVL leading edge. 4 μm width stacks were captured every 30 s. Maximum projection is shown. Scale bar 25 μm. Red arrow points to E-YSL. The photo-bleached membrane (blue circle) is not removed or endocytosed and remains unchanged up to its enclosure within the advancing E-YSL.

Supplementary Movie 3 Endocytosis impairment in *rab5ab* YMOs. Confocal video time-lapse of control and *rab5ab* YMOs (medium dose) soaked in Rhodamine B-Dextran for 5 minutes at sphere stage. 20 μm width stacks were captured every 4 min. Scale bar 25 μm. Note the reduced dextran internalization ahead of the advancing EVL.

Supplementary Movie 4 Medium dose *rab5ab* YMO macroscopic phenotype. Macroscope video time-lapse of control and *rab5ab* YMO sibling embryos (at a middle dose) are shown. Bright field images were captured every 5 min from sphere stage to 16 HPF. Scale bar 250 μm. Note that the *rab5ab* YMO at a middle dose is at 60% epiboly at the time when the control embryo closes (yellow arrow). The *rab5ab* YMO continues its elongation in the animal-vegetal direction but remains open.

Supplementary Movie 5 High dose *rab5ab* YMO macroscopic phenotype. Macroscope video time-lapse of control and *rab5ab* YMO sibling embryos (at a high dose) are shown. Bright field images were captured every 5 min from sphere stage to 16 HPF. Scale bar 250 μm. White arrows point to the leading front, the red arrow highlights the folding of the DCs layer and the asterisk denotes the space generated in between the DCs and the internal part of the yolk. Note that *rab5ab* YMO at a high dose halt before reaching the equator and burst.

Supplementary Movie 6 Cortical Myosin flows on the E-YSL in control versus *rab5ab* YMOs. Spinning-disk video time-lapses of zebrafish transgenic [Tg (β-*actin:MYL12.1-eGFP*)] embryos from 50% epiboly onward (control sibling (top) and a *rab5ab* YMO (bottom). The movements of cortical actin were analyzed by Particle Image Velocimetry (PIV). 9 μm width stacks were captured every 12 s. Maximum projections are shown. Scale bar 25 μm. The retrograde animalward cortical myosin flows from the vegetal pole of the yolk cell toward the E-YSL are impaired in *rab5ab* YMOs.

Supplementary Movie 7 Enveloping layer (EVL) marginal cells elongate in the CC direction in *rab5ab* YMOs. Confocal video time-lapse of control and *rab5ab* yolk cell membrane labeled [Tg (β*-actin:m-GFP*)] zebrafish transgenic YMOs (medium dose). Scale bar 25 μm. While control embryos marginal EVL cells elongate in the animal/vegetal direction (left), *rab5ab* YMOs marginal cells flatten and elongate in the circumferential direction. The speed of progression of the EVL is reduced and it eventually stalls.

Supplementary Movie 8 Yolk granules flow lines in *rab5ab* YMOs. Flow trajectories from PIV measurements of a two-photon video time-lapse of a membrane-GFP [Tg (β-*actin:m-GFP*)] zebrafish transgenic *rab5ab* YMO at the middle plane, 350 μm deep inside, throughout epiboly. Scale bar 100 μm. Flows in the *rab5ab* YMO are partly arrested.

Supplementary Movie 9 Yolk granules kinematics in *rab5ab* YMOs. PIV measurements of two-photon excitation stitched video time-lapses of membrane-GFP [Tg (β-*actin:m-GFP*)] zebrafish transgenic Control (left) and *rab5ab* medium dose (center) and high dose (right) YMO siblings at the middle plane, 350 μm deep inside, throughout epiboly. Scale bar 100 μm. Time in hours. The *rab5ab* YMOs show epiboly delay and disrupted yolk flows. They eventually burst at high dose.

Supplementary Movie 10 Power Density Maps in *rab5ab* YMOs. Two-photon excitation video time-lapse of a membrane-GFP [Tg (β-*actin:m-GFP*)] *rab5ab* YMO at the middle plane, 350 μm deep inside, throughout epiboly (left). Mechanical power density maps were calculated by HR from the velocity fields, color-coded at an equal scale for each time point (right) and overlaid on the fluorescence images (negative - blue; 0 - green; positive - red) (middle). The relative mean square error for the Power is displayed as a percentage for each time point. Scale bar 100 μm. As in the wild type (Hernandez-Vega et al., 2017), during doming, most of the energy supply is generated by the blastoderm. After 50% epiboly, the E-YSL constriction becomes the main motor of epiboly, while the EVL cells resist their stretching.

Supplementary Movie 11 Comparison of Power Density Maps between *rab5ab* and Control YMOs. Mechanical power density maps (discrete power level tracks representation) obtained by HR analysis from the velocity fields over time. Maps were, color-coded at an equal scale for each time point and overlaid on the fluorescence images (negative - blue; 0 - green; positive - red) (middle). Qualitatively, *rab5ab* YMOs display no spatial differences with Control YMOs at early times up to 55% epiboly although their power density values are considerably weaker. They also reach after 70% epiboly a final equivalent distribution but take much longer to reach this developmental stage. Times are in hours. Scale bar 100 μm.

Supplementary Movie 12 Longitudinal and latitudinal stresses in *rab5ab* YMOs. Main surface stresses were calculated by HR of velocity fields obtained from stitched two-photon excitation video time-lapse of a membrane-GFP [Tg (β-*actin:m-GFP*)] *rab5ab* YMO embryo at the middle plane, 350 μm deep inside, throughout epiboly. Longitudinal (red) and latitudinal (green) stresses and their differences (blue) were plotted as a function of the φ angle from animal to vegetal. Time points were every 40 min. The equator - dotted yellow line -, yolk cell surface - purple shadow - and the relative mean square error of the pressure as a percentage for each time point are displayed. In these YMOs, the longitudinal and latitudinal stresses are equal at the poles. Contrary to wild type embryos, the latitudinal stress does not steep up from animal to vegetal. Their difference (longitudinal minus latitudinal) shows a defined profile at the EVL margin after 50% epiboly, becoming positive in the EVL and negative at the E-YSL.
