# Supplementary figures and images for: Rab5ab-Mediated Yolk Cell Membrane Endocytosis Is Essential for Zebrafish Epiboly and Mechanical Equilibrium During Gastrulation
Source: Front Cell Dev Biol. 2021 Oct 29;9:697097. doi: 10.3389/fcell.2021.697097 (PMC8585776; doi:10.3389/fcell.2021.697097)

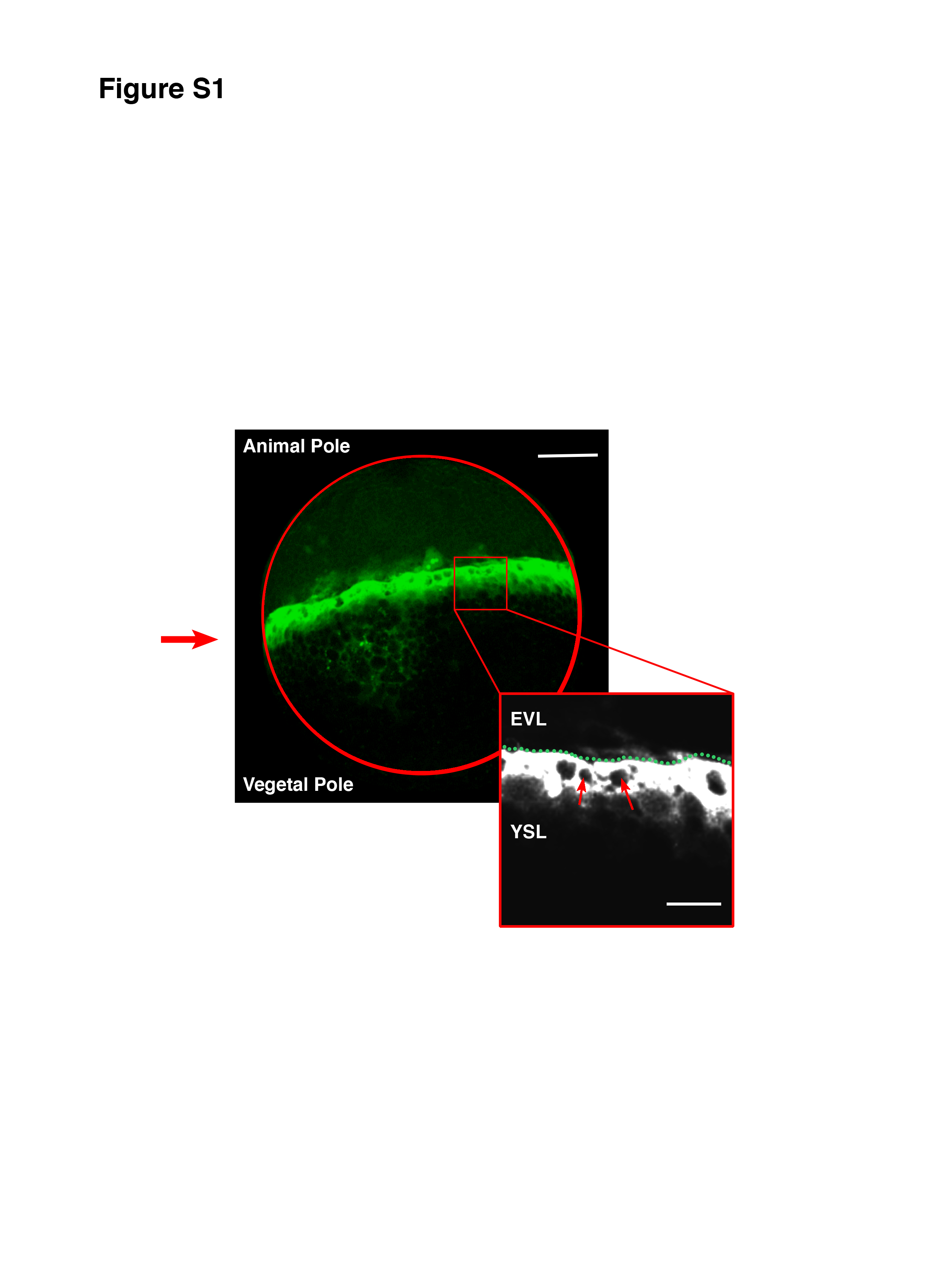

Supplement: Supplementary Figure 1 — Yolk Cell-Specific Morphants (YMOs). Fluorescent image of an embryo injected with a fluorescently labeled Morpholino (FITC) at the 512 cells stage and observed at 40% Epiboly. The Animal and Vegetal Pole are indicated. The red arrow points to the fluorescence in the yolk cell, accumulating at the E-YSL. Scale bar 200 μm. The inset shows at high magnification the area in which the labeled morpholino was incorporated (E-YSL) adjacent to the EVL (delimited by a green dotted line). Arrows point to the YSL nuclei which do not incorporate the injected morpholino. Scale bar 25 μm. [file Image_1.TIF]

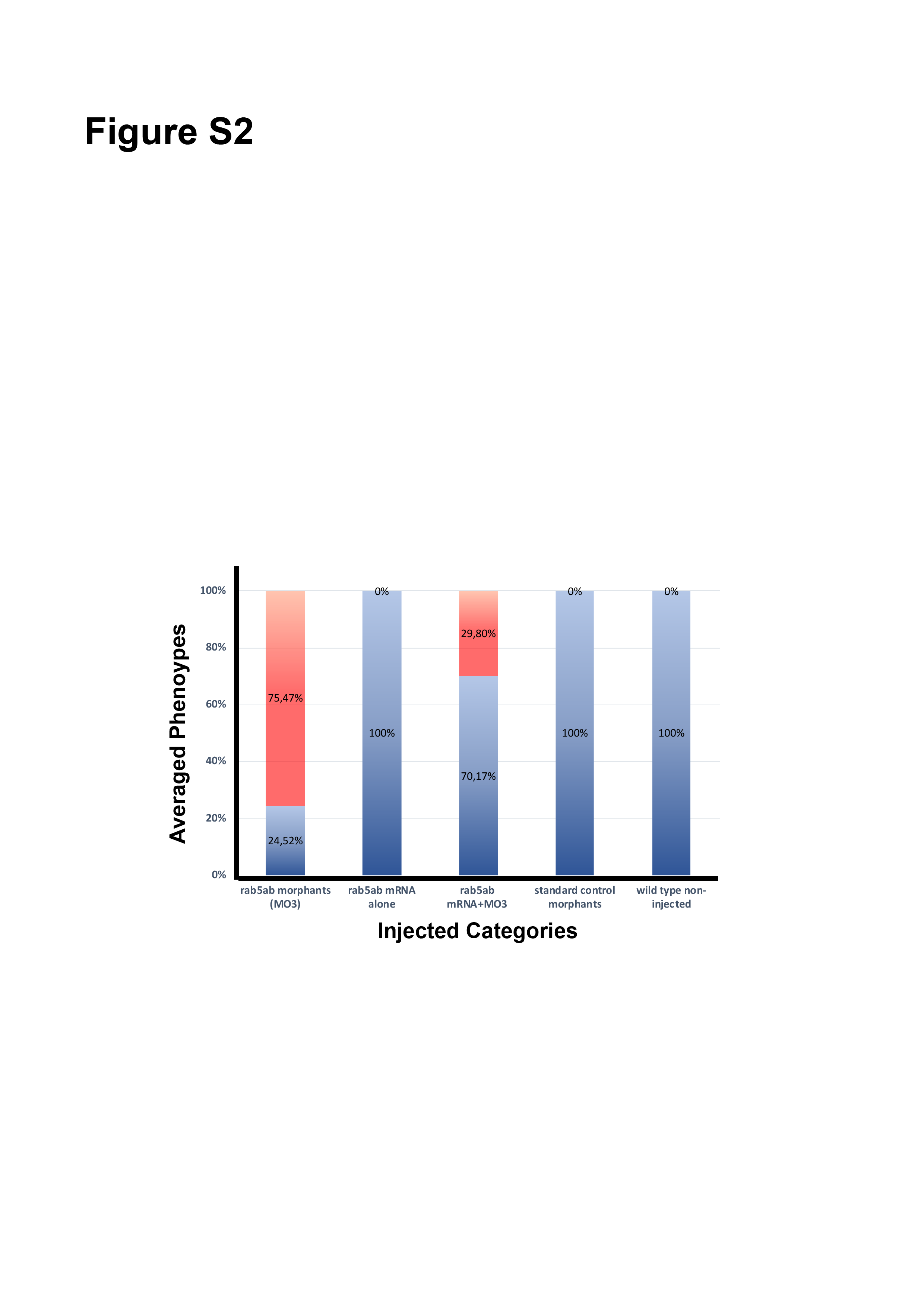

Supplement: Supplementary Figure 2 — rab5ab YMO rescue. The phenotype of rab5ab YMO embryos (MO3) (n = 53), embryos injected with rab5ab mRNA alone (n = 48), rab5ab YMO + rab5ab mRNA injected embryos (n = 57) and standard Control YMO embryos (n = 47) were evaluated by comparison to wild-type non-injected embryos (n = 50) at shield - 70% epiboly stage. The result is represented as the percentage of normal (blue bar) and abnormal (red bar) embryos in each group. [file Image_2.TIF]

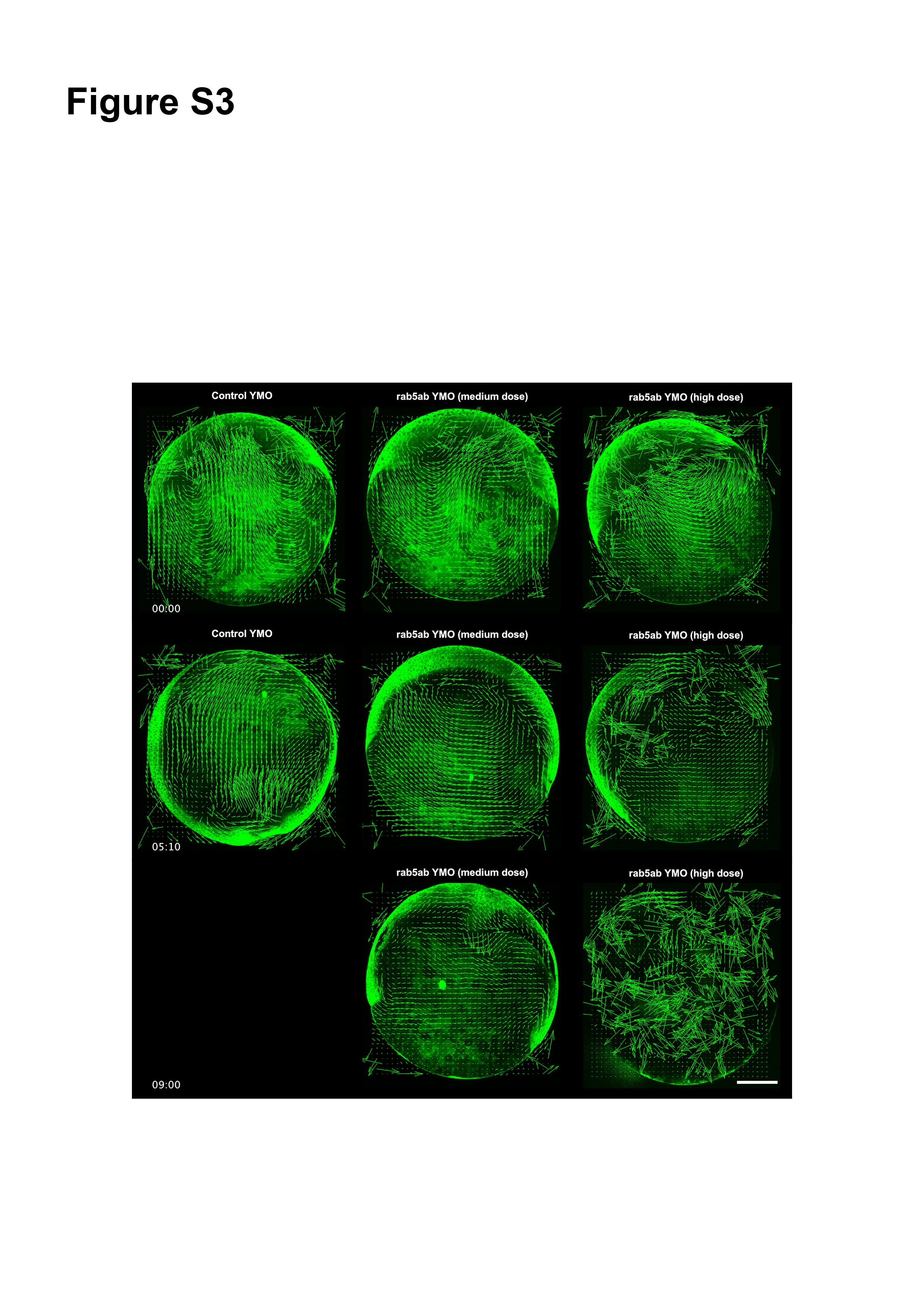

Supplement: Supplementary Figure 3 — Yolk granules flows patterns in rab5ab YMOs. PIV of time-lapse snapshots imaged by two-photon microscopy Control and rab5ab YMO (medium and high dose) embryos (from Supplementary Movie 9). Meridional sections (350 μm depth from the yolk cell membrane surface) were collected from [Tg (β-actin: m-GFP)] embryos. While Control embryos undergo epiboly on time, medium dose rab5ab YMOs delay and their yolk granules flow patterns are altered. High dose rab5ab YMOs eventually burst. Scale bar 200 μm. Time in hours. [file Image_3.TIF]

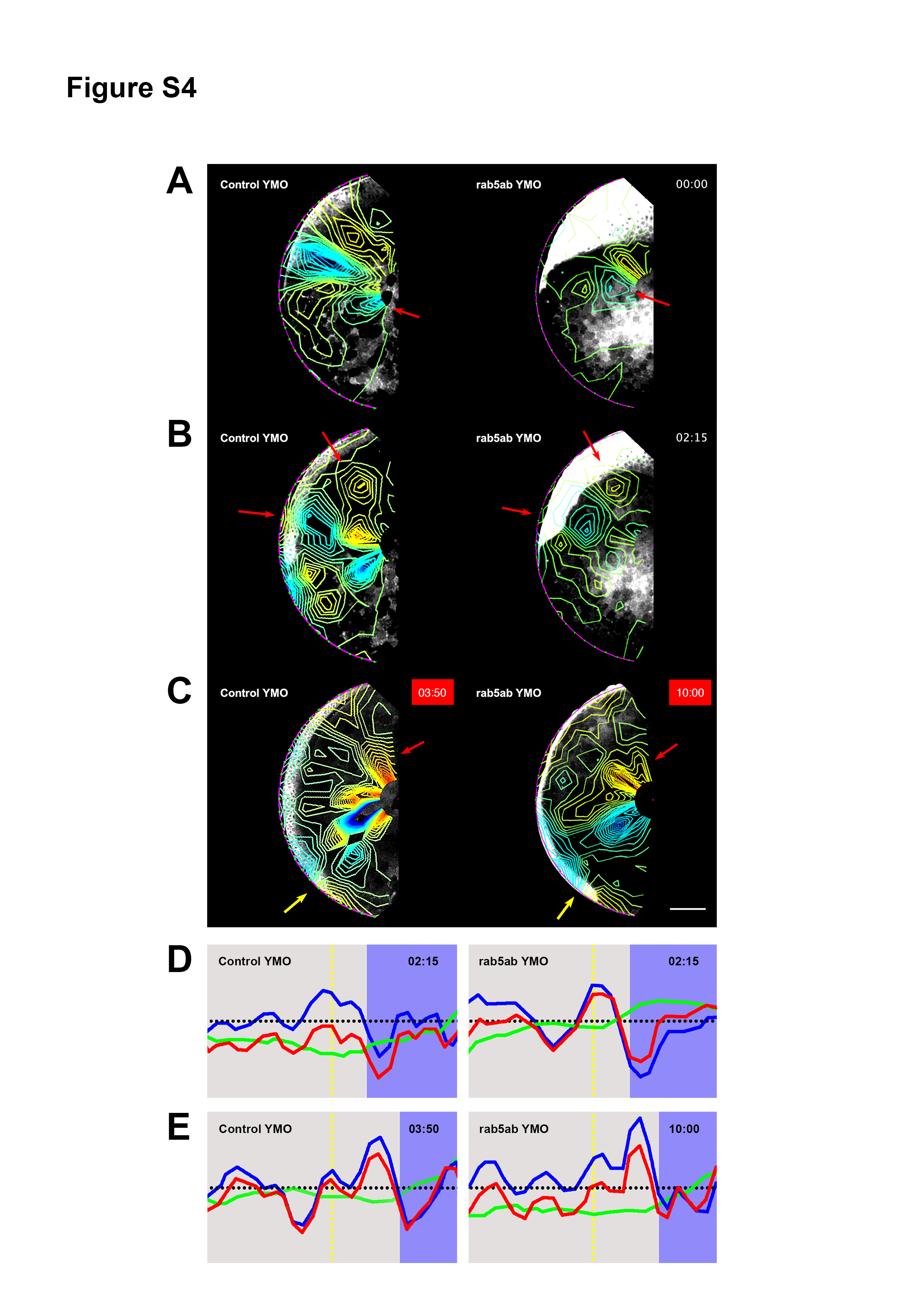

Supplement: Supplementary Figure 4 — Comparison of the mechanical properties of Control and rab5ab YMOs. (A–C) Snapshots of sequential times of Control and medium dose rab5ab YMOs at different times of development (from Supplementary Movie 11). Mechanical power density maps (discrete power level tracks representation) obtained by HR analysis over time. Qualitatively, rab5ab YMOs display no differences with Control YMOs in the spatial distribution of mechanical work at early times and at 55% epiboly (red arrows). They also reach a final equivalent distribution although at weaker levels (yellow arrows) after 70% epiboly. rab5ab YMOs take much longer to reach this developmental stage than Control YMOs Red shaded timing. Times are in hours. Scale bar 100 μm. (D) and (E) Longitudinal (red) and latitudinal (green) stresses and their differences (blue) along the embryo cortex at two different times (55% and 75% epiboly) in membrane-GFP transgenic [Tg (β-actin:m-GFP)] Control and rab5ab YMO as in Figure 4. The equator - dotted yellow line - and yolk cell surface - purple shadow - are displayed. The latitudinal stress does not steep up from animal to vegetal in rab5ab YMO until 70% epiboly at a much later time that Control YMOs. Times are in hours. [file Image_4.TIF]

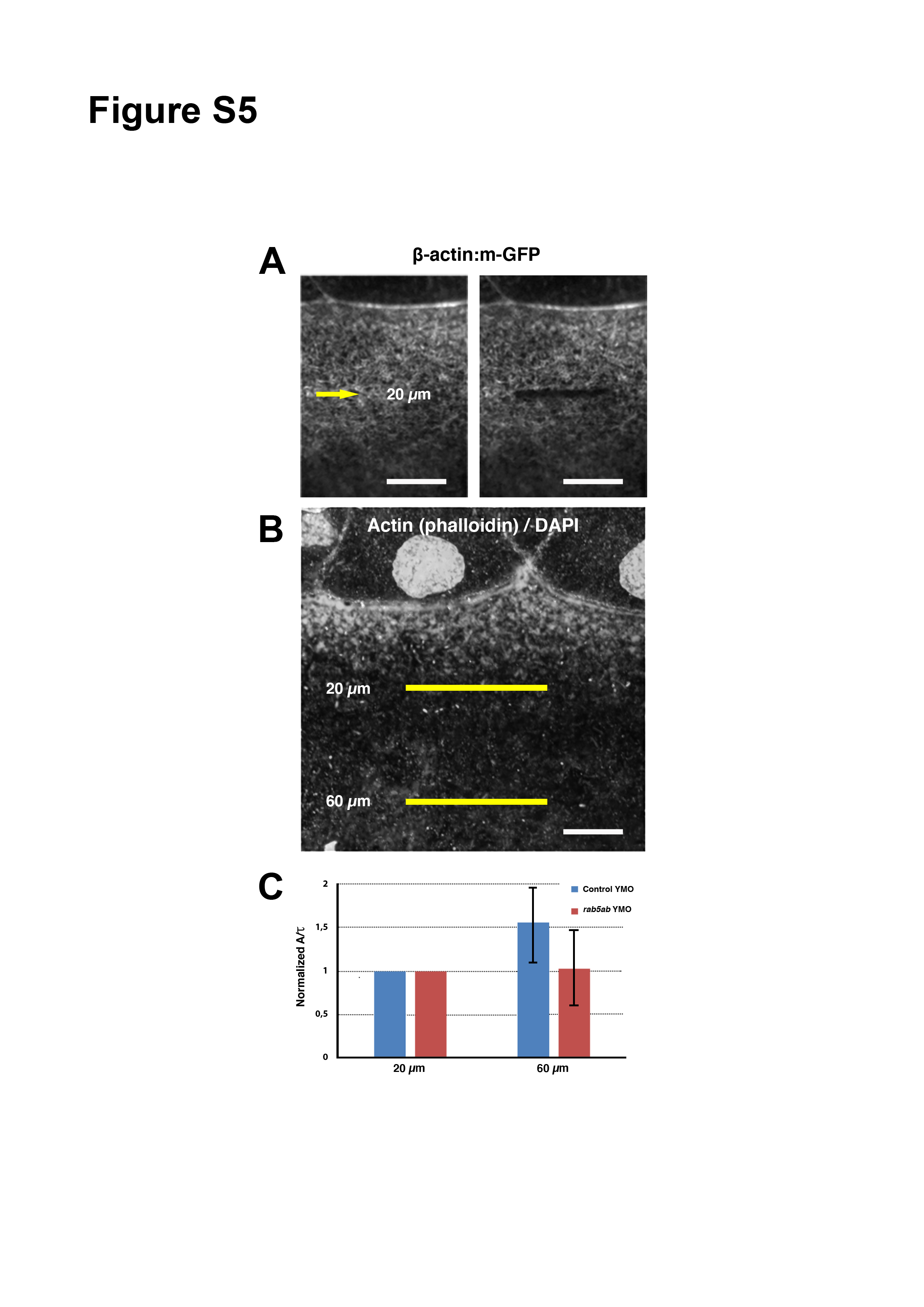

Supplement: Supplementary Figure 5 — Animal/Vegetal gradient of Tension: laser microsurgery. (A) Laser surgery of the actomyosin cortex was performed with a pulsed UV laser (355 nm, 470 ps per pulse) by inducing plasma-mediated ablation. To compare the cortical tension in the AV directions, a 20 μm laser line containing 50 pulses was scanned five times at a frequency of 800 Hz, parallel the EVL front, centering the cut at a distance of about 20 μm. Scale bar 10 μm. (B) This laser ablation regime (same laser line length) was employed at 20 and 60 μm to study the surface gradient of tension in rab5ab and Control YMOs at 65% epiboly). Scale bar 10 μm. (C) To quantify the AV surface gradient of tension, we normalized the recoil velocities at each distance from the EVL margin to the recoil velocity at 20 μm distance and tested the statistical significance of the gradient using the Wilcoxon rank sum test. The gradient does not develop in rab5ab YMOs (P < 0.05) when comparing to Control YMOs. [file Image_5.TIF]
